# Diagnostic Images of the Ocular Fundus Using a Low-Cost Portable Endoscope in Premature Patients at Risk of Developing Retinopathy of Prematurity

**DOI:** 10.1155/2020/5864565

**Published:** 2020-06-09

**Authors:** Luz Consuelo Zepeda-Romero, Jose Alfonso Gutierrez Padilla, Daniel Perez-Rulfo Ibarra, Jaime Orozco-Perez, Rodrigo Alonso Delgadillo, Lilia Paulina Rodríguez, Guillermo Yanowsky-Reyes

**Affiliations:** ^1^Oftalmologia Neonatal, Cirugia Pediatrica, Hospital Civil de Guadalajara Fray Antonio Alcade, Guadalajara, Jalisco, Mexico; ^2^Centro Universitario de Ciencias de la Salud, Universidad de Guadalajara, Guadalajara, Jalisco, Mexico; ^3^Jefatura de enseñanza, Secretaria de Salud Jalisco, Gobierno del estado de Jalisco, Guadalajara, Mexico; ^4^Teaching coordinator in Pediatrics PNPC Conacyt, Hospital Civil de Guadalajara, Guadalajara, Mexico; ^5^Cirugia Pediatrica, Hospital Civil de Guadalajara Fray Antonio Alcade, Guadalajara, Mexico

## Abstract

The purpose of this article is to describe how fundus images are obtained using a low-cost device: the “Visual Ear Wax Cleaner Tool” portable endoscope (Soonhua Inc., China) connected to a smartphone, after installation of free applications (“Inskam” and “CameraFI”) using the smartphone screen as a monitor and after medication mydriasis, local anesthesia, and blepharostat placement. With this endoscope, video recording and fundus imaging are easily performed, for the case of patients at the risk of developing retinopathy of prematurity (ROP), facilitating timely screening in order to start treatment in patients who require it. This fundus imaging technique shares certain similarities with the RetCam® (Clarity, Pleasaton California) system, which performs real-time fundus imaging providing the ability to record and document findings and capture images from the video footage, with high quality and definition, although with a smaller angle of vision. The capture of images using a smartphone allows storing and sharing the images. These are devices which are generally accessible and portable and which use simplified energy sources, requiring very simple training. The low-cost, easy-to-learn technique and quick sharing of images through communication networks make this a tool to be considered for the practice of telemedicine.

## 1. Introduction

Retinopathy of prematurity is a potentially serious disease that causes a vascular development disorder in the retina of premature newborns with incomplete retinal vascularization. It is the main cause of preventable childhood blindness in developing countries. In most cases, its appearance is associated with a poorly controlled administration of oxygen in neonatal care units.

In low- and middle-income countries, the prevalence of the disease varies greatly from one country to another and even from one neonatal intensive care unit to another in the same country. It can affect up to 34% of premature babies weighing less than 1500 grams at birth, of which 6 to 27% will require treatment [1].

Preventing blindness caused by this disease depends on its timely detection, and thus accessible technology and easy handling can prevent a large number of cases of childhood blindness [1] Photography of the retina (ocular fundus) is among the many advances made in relation to this disease and others in the field of ophthalmology. It is a fundamental tool in the daily practice of ophthalmology but requires sophisticated and specialized instruments that can be expensive, costing up to hundreds of thousands of dollars, as for the RetCam (Clarity, Pleasanton California) [2].

The objective of this report is to describe the feasibility of taking eye fundus pictures and screening for posterior pole ROP findings with a low-cost endoscope. Devices such as this endoscope, once validated, can be a valuable tool for the practice of telemedicine which would help the screening of newborns at risk that need to be referred to an ophthalmologist for diagnosis and timely treatment [3].

Telemedicine using RetCam has shown good results when identifying newborns at risk of suffering from this disease [4].

There is an urgent need for resources that are easily accessible and inexpensive and can be easily used by the healthcare personnel other than ophthalmologists for the timely detection of ROP and timely management of each patient at risk [5].

The history of ophthalmological photography dates back to 1800 when Jackman and Webster described a technique for photographing a person's retina. In 1950, the electronic flash and 35 mm cameras were adapted to be used as ophthalmological instruments, and thus photography in ophthalmology was born [6].

Since 1997, there is technology available to document fundus findings in newborns: the RetCam® wide-field imaging system. It delivers high-quality images, but with a high-cost limitation.

The RetCam® imaging system is a wide-field camera (up to 130°) that is used to capture images of the retina of premature infants. It was specially designed for pediatric use, employing state-of-the-art digital technology that captures images in high resolution and records videos quickly and easily, even in noncollaborating patients, cost and portability being its main limitations [7].

Similarly, since 2012, smartphones have been used with 20, 28, 30, 40, and 60 *D* ophthalmic lens, to examine the fundus, and are safe for use in humans [8, 9].

In recent years, multiple options for pediatric digital photography have been developed, some of them offering wider field of view and relatively low costs [10, 11].

## 2. Materials and Methods

Institutional Review Board of the Hospital Civil de Guadalajara approval and parent consent were obtained; funduscopic images were taken with a portable endoscope. In this case, we used the “Visual Ear Wax Cleaner Tool” (Soonhua Inc, China), connected to a “Motorola One” smartphone, using the “Inskam and “CameraFi” (Vault Micro, Inc.)” applications. These applications (apps) are necessary to link the endoscope to the mobile phone's camera (Android, Mac, or Windows) and thus obtain recorded video and/or photographs in real time with excellent quality.

This fundus video-recording technique using a portable endoscope and a smartphone involves multiple steps, which are described below. The technique is simple; however, it can take a few attempts to master it as the user must learn to position the endoscope correctly and with proper orientation as well as adjust the intensity of the light (6 LED lights). In addition, it is necessary to use a blepharostat, and some practice and knowledge is required. Good pupillary dilation before placement of the endoscope is of the utmost importance, as is applying topical mydriatic medication (phenylephrine and tropicamide, Sophia Lab Mexico), local anesthesia (tetracaine, Sophia Lab, Mexico), and hypromellose 2% (Sophia Lab, Mexico) for proper exploration and visualization of the fundus.

**Figure 1:**
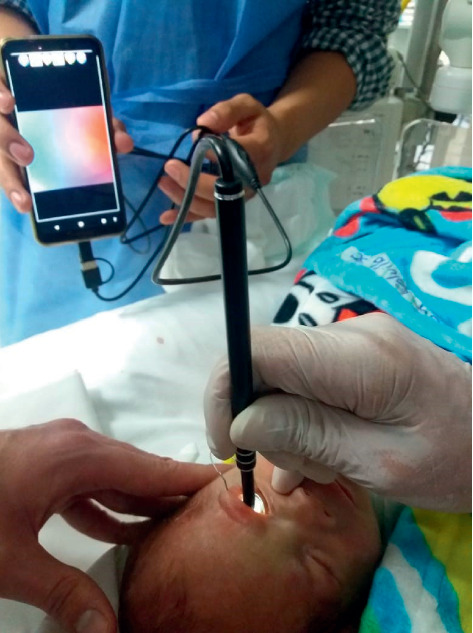
Right eye fundus exploration in a nonsedated preterm infant using the portable endoscope.

Drops were administered, the blepharostat placed, and hypromellose 2% ophthalmic gel applied. A disposable contact lens is used over the cornea to protect it from direct contact with the “Visual Ear Wax Cleaner tool” endoscope previously connected to a smartphone. Using gentle movements, recording of the eye fundus is initiated, and the intensity of the LED light can be adjusted as necessary (Figure 1).

After recording the video, the app can store it on the phone and convert it into MP4 and jpg formats, facilitating its reproduction, selection of images, and communication of the findings. The ease of dissemination of the acquired data should be highlighted, facilitated by direct storage on the mobile device which does not require any other applications to access such information.

It is very important to maintain the confidentiality of all personal data in accordance with the Data Protection Act of 1998 and the Access to Health Records Act of 1990. The patient's legal guardians shall previously sign an informed consent for the procedure, and their data are stored as confidential in the hospital's internal digital system where the patient is hospitalized.

## 3. Results and Discussion

Eye fundus images and videos of one eye of 20 premature patients' (BGA 28–34 weeks, BW 740–1800 g) were successfully obtained by a pediatric ophthalmologist, using the portable endoscope camera, “Visual Ear Wax Cleaner Tool” (Soonhua Inc, China) which has an adjustable 6-LED light source, connected to a “Motorola One” smartphone; findings in zone 1 and 2 as pre-plus, plus disease, and hemorrhage are easily identified; stages 1-2 ridges out of this area are difficult to capture (Table 1).

Smartphones are increasingly useful not only for communication purposes but also for other applications in healthcare, which is why they are being used increasingly in ophthalmology to document the characteristics of the pathologies suffered by patients. Other techniques used to perform patient assessment and screening, though effective, require specialized instruments and are difficult to access due to their high cost. Therefore, the technique described in this paper is a new option to perform the same activity but with low-cost tools that are easily accessible and that any healthcare personnel can use due to its ease of use and minimal training required.

**Figure 2:**
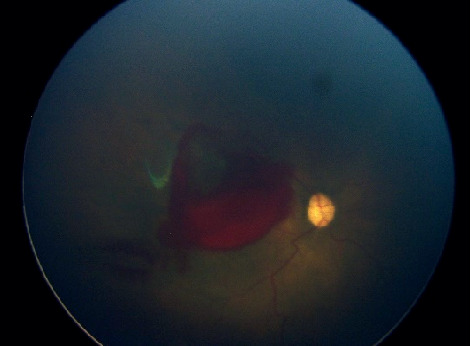
RetCam right eye fundus photograph of a premature patient with 33.5 weeks of postmenstrual gestational age with ROP treated with antiVEGF, which developed a macular hemorrhage.

**Figure 3:**
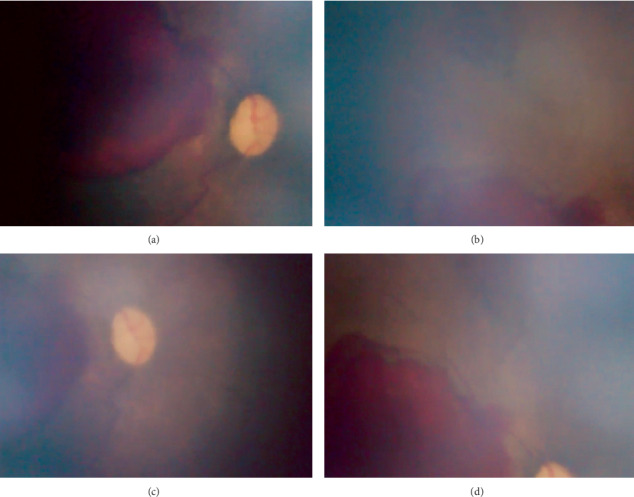
ABCD photographs of the same premature patient with 33.5 weeks of gestation taken with a portable endoscope camera, “Visual Ear Wax Cleaner Tool.”

**Figure 4:**
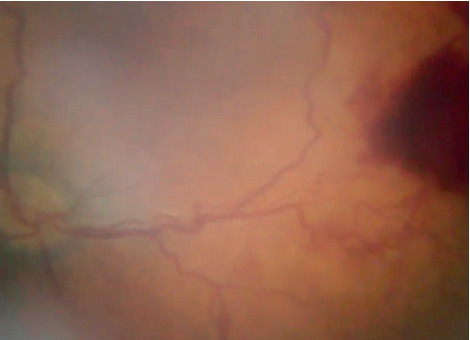
Left eye, right fundus of a 34-week-old postmenstrual age female, with aggressive posterior retinopathy.

**Figure 5:**
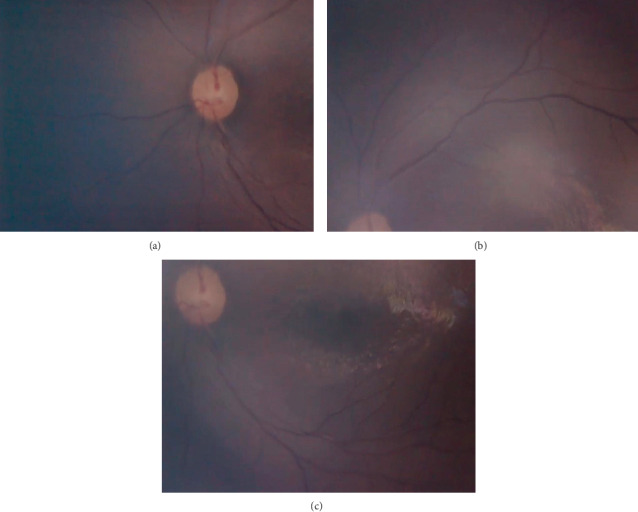
(a–c) Pictures of nasal and temporal superior and inferior retina, zones 1 and 2.

**Figure 6:**
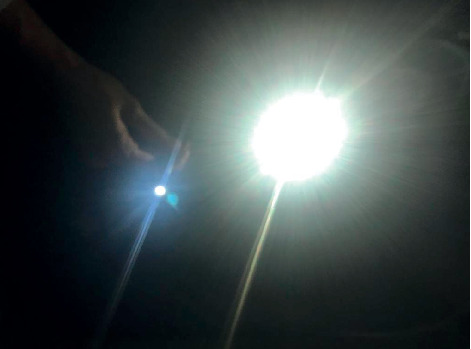
Light emitted by the endoscope and iPhone 6 LEDs.

Even with a relative limited field of view (70 degrees of retina) compared with RetCam (Figure 2), eye fundus swept can be done to take different angles (Figures 3(a)–3(d)); endoscope displacement can be done to take different eye fundus angles, or a central single view can indentify the presence of plus diseace(Figures 4 and 5(a)–5(c)). The aforementioned portable endoscope is a highly functional and accessible option for the trained health personnel in need of screening premature infants. The main advantages of this examination technique include its low cost (20 US dollars), its portability due to its small size, simple operability, high-resolution images, and possibility of easily documenting and sharing the findings. Additionally, it allows for regulating the intensity of the light source, which does not generate heat as it uses LED light. It is known that the amount of light intensity used should not exceed safety limits described by ISO 15004-22; the light emitted by the endoscope LED's has not been measured, which is subjectively less than that emitted by iPhone 6 (Figure 6) [12]. Among the features that could be improved in the camera presented in the endoscope are the diameter, a wider camera area (7-8 mm), a condensing lens that could provide a greater field of view, and a disposable optical-quality protective accessory to reduce the potential risk of corneal abrasion and infection during the exploration, keeping all of them with High quality and low cost materials.

## Figures and Tables

**Table 1:** Comparison of clinical fundus findings identified with BIO binocular indirect ophthalmoscope and portable endoscope.

Patient No. Laterality	Findings with binocular indirect ophthalmoscope	Findings using the portable endoscope
1 LE	Vessels developed to ora serrata	Vascular development present at zones 1 and 2, no plus disease
2 LE	ROP 1 at zone 3, no plus disease	Vascular development present at zones 1 and 2, no plus disease
3 RE	Avascular temporal at zone 3, no ridge, no plus disease	Vascular development present at zone 1 and 2, no plus disease
4 LE	No plus disease, vascular development at zone 2, avascular at zone 3	No plus disease, vascular development at zone 2
5 RE	ROP treated with antiVEGF, pre-plus disease, hemorrhage in macular area, avascular at zones 2 and 3	ROP treated with antiVEGF, pre-plus disease, hemorrhage in macular area, avascular at zone 2
6 RE	Avascular temporal at zone 3, no ridge, no plus disease	Vascular development present at zones 1 and 2, no plus disease
7 RE	No plus disease, vascular development at zone 2, avascular at zone 3	No plus disease, vascular development at zone 2
8 LE	No plus disease, vascular development at zone 2, avascular at zone 3	No plus disease, vascular development at zone 2
9 RE	Retinal vessels developed to ora serrata, no plus disease	Vascular development present at zones 1 and 2, no plus disease
10 LE	Type 1 ROP, posterior aggressive ROP	Type 1 ROP, Plus disease, and hemorrhage at zone 1
11 RE	Retinal vessels developed to ora serrata, no plus disease	Vascular development at zones 1 and 2, no plus disease
12 LE	Retinal vessels developed to ora serrata, no plus disease	Vascular development at zones 1 and 2, no plus disease
13 LE	No plus disease, vascular development at zone 2, avascular at zone 3	No plus disease, vascular development at zone 2
14 RE	Avascular temporal at zone 3, no ridge, no plus disease	Vascular development present at zones 1 and 2, no plus disease
15 RE	Retinal vessels developed to ora serrata, no plus disease	Vascular development present at zones 1 and 2, no plus disease
16 RE	No plus disease, vascular development at zone 2, avascular at zone 3	No plus disease, vascular development at zone 2
17 LE	Avascular temporal at zone 3, no ridge, no plus disease	Vascular development present at zones 1 and 2, no plus disease
18 RE	Type 1 ROP stage 3, zone 2, with plus disease	Type 1 ROP stage 3, zone 2 with plus disease
19 LE	No plus disease, vascular development at zone 2, avascular at zone 3	No plus disease, vascular development at zone 2
20 LE	Avascular temporal at zone 3, no ridge, no plus disease	Vascular development present at zones 1 and 2, no plus disease

RE: right eye; LE: left eye

## Data Availability

The data used to support the findings of this study are available from the corresponding author upon request.
